# Body Posture and Low Back Pain: Differences between Folk and Ballroom Dancers

**DOI:** 10.3390/healthcare12020137

**Published:** 2024-01-08

**Authors:** Maciej Kochman, Gabriela Cmela, Wojciech Kasperek, Agnieszka Guzik, Mariusz Drużbicki

**Affiliations:** Physiotherapy Department, Institute of Health Sciences, College of Medical Sciences, University of Rzeszów, 35-215 Rzeszów, Poland

**Keywords:** low back pain, posture, spine curvatures, spine mobility, dance, physical activity

## Abstract

(1) Background: Dance is extremely diverse in its styles. Each of them presents different training, dynamics, and figures that may impact the body posture and the occurrence of low back pain. This observational study aimed to compare the sagittal curvatures and the range of motion (ROM) of the spine, as well as the low back pain occurrence and its intensity between folk and ballroom dancers. (2) Methods: Fifty-one participants took part in the study (nineteen folk dancers, fifteen ballroom dancers, and seventeen non-dancers) aged 18–32. Study groups did not differ in anthropometric parameters as well as in dancing experience and training frequency. Study procedures included a self-administered questionnaire and a physical examination of the sagittal spine curvatures and ROM. The questionnaire included questions about epidemiological data and the occurrence of chronic pain and its intensity using a Visual Analogue Scale (VAS). (3) Results: There was a significant difference in thoracic kyphosis angle between study groups (*p* = 0.02). The greatest angle was found in folk dancers and the lowest in ballroom dancers (40 vs. 33 respectively). We have found no significant differences in spine ROM, low back pain occurrence, and intensity between study groups (*p* > 0.05). We have found no correlation between low back pain and spine curvatures and ROM in dancers (*p* > 0.05), however, we found a very strong and negative correlation between thoracic spine range of motion and the pain intensity in non-dancers (R= −0.95, *p* = 0.003). The analysis also revealed that only in folk dancers, but not in ballroom dancers, the BMI correlates positively with dancing experience (R = 0.67, *p* = 0.002). (4) Conclusions: There are no differences in low back pain occurrence and pain intensity between folk and ballroom dancers, however, the prevalence of low back pain in dancers is very high. Folk dancers seem to have more flexed body posture compared to ballroom dancers.

## 1. Introduction

Dance is an art that is extremely diverse in its styles. Along with physical fitness, including strength, flexibility, balance, limb coordination, endurance, and motor control, dance is an aesthetic form of artistic expression [1,2,3]. Each dance style presents different dynamics and figures, which may have a different impact on the frequency, types and severity of musculoskeletal overuse disorders [4,5]. For this reason, it can also be suspected that dancers of particular styles may be distinguished by their specific body shape.

Polish folk ensembles are artistic groups whose repertoire is based on traditional folk culture, presenting it through dance and singing [6]. The folk dance is a sign of ethnic identity, ancestral legacy and rituals deeply rooted in national traditions [7]. Polish folk ensembles include regional groups, created by amateurs, striving for authenticity, and stylized ensembles, incorporating acrobatic and ballet elements into their dance [8]. Typically, folk dance choreographies are based on folklore and then developed and made more attractive by using creative and spectacular elements [9]. Ballroom dancing is one of the disciplines that develop various motor skills. It requires precise spatial and temporal coordination with postural control that allows dancers to synchronize their body movements with the music [10,11]. The aesthetics of dancing require precise matching from partners [12]. The characteristic and often used closed-hold position, in which the dancers’ upper body segments are connected, requires the partners to coordinate their movements [13]. From a biomechanical perspective, many forces are acting on bodies in a dancing couple, which can make controlling movements more difficult than when dancing solo [13,14]. Moreover, maintaining such a posture for a long time requires great stability and trunk control of both partners [14,15]. Ballroom dancers, especially females, are often characterized by a slender silhouette, low body weight, and low body fat, which of course may be influenced by the training process requiring high energy expenditure, but may also be affected by high personal standards and desiring low body fat as it is believed to improve performance [16,17,18]. Due to the intensive dance training and complex movement involving jumps, turns and rapid changes in direction, dancers often suffer musculoskeletal injuries and low back pain [19]. The pathogenesis of low back pain in dancers is, however, multifactorial and remains non-specific [19,20]. Some studies suggest that low back pain in dancers may be linked to the postural adjustments resulting from dance practice [19,21,22]. This relationship is, however, poorly explored [21,22]. We reviewed the literature, but we did not find any study that would conduct a comparative analysis of Polish folk and ballroom dancers, which was the inspiration to undertake this research. When comparing reports, we noticed a clear disproportion in the availability of studies regarding various forms of dance [23,24,25] as most of them focused on ballet [26], fewer studies could be found on ballroom dancing [27] and the least studies concerned folk dancing [28], especially Polish one. To the best of the authors’ knowledge, there are very limited reports in the scientific literature assessing the impact of long-term dance practice on folk dancers’ body posture as well as mobility of spine and low back pain. In this study, we focused on comparing the body posture, mobility of spine and low back pain in ballroom and Polish folk dancers. Both of these styles are couple dances, which particularly affect the biomechanics of two partners and require exceptional coordination of the movements of both dancers. Apart from that, they are very different from each other; Polish folk dance is more squat and requires a lot of jumping, kneeling, spinning, and maintaining the immobile trunk; ballroom dancing, on the other hand, often requires high movement dynamics, a greater hyperextension of the spine and rotation movements of the trunk and pelvis. This study aimed to assess the impact of practicing Polish folk and ballroom dancing on the dancers’ body posture as well as spine mobility and low back pain. We asked the following research questions: (1) Are there differences in spine mobility and the depth of spine curvatures between folk, ballroom dancers and non-dancers? (2) Are there differences in the occurrence and intensity of low back pain in the study groups? (3) Are there any correlations between the intensity of low back pain, spine mobility and depth of spine curvatures in the study groups? (4) Are there any correlations between dance experience and training intensity with pain intensity and BMI in the study groups?

## 2. Materials and Methods

### 2.1. Study Participants

**Figure 1:**
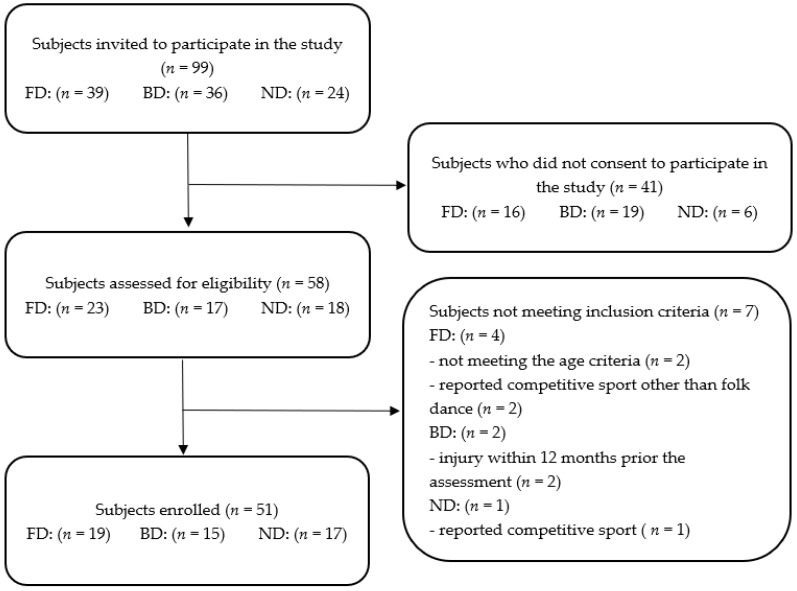
Study participants’ flowchart.

For this observational study, we invited ninety-nine participants living in south-eastern Poland: thirty-nine Polish folk dancers (FD), thirty-six ballroom dancers (BD), and twenty-four non-dancers (ND). Apart from dancing, the participants were high school/university students or white-collar workers. Forty-one participants did not consent to the study so fifty-eight participants were assessed for the eligibility criteria. The inclusion criteria were as follows: (1) given written informed consent to participate in the study, (2) age 18–32, (3) at least 2 years of folk/ballroom dancing experience (dancing groups only), (4) lack of concomitant diseases, lower limbs, and spine injury or surgery within 12 months prior the assessment, or acute inflammation. The exclusion criteria were as follows: (1) lack of written informed consent, (2) age below 18 or above 32, (3) confirmed lower limbs or spine injuries and surgeries within 12 months prior to the assessment, (4) performing competitive physical activity (non-dancers group) or performing competitive physical activity other than folk or ballroom dancing (dancing groups only), (5) performing physical work for a living. Due to eligibility criteria, seven participants were excluded from the study (four FD, two BD and one ND). Finally, fifty-one participants (thirty females and twenty-one males) aged 18–32 were included in the study. The study groups consisted of nineteen FD, fifteen BD and seventeen ND matched to the study dance groups for gender and age. Participants’ flowchart has been shown in Figure 1.

### 2.2. Study Procedures

**Figure 2:**
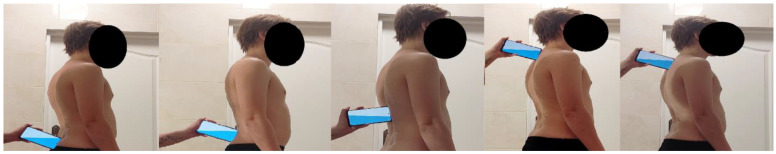
The measurement of the spinal curvatures.

All study procedures were conducted from November 2022 to May 2023. The participants were recruited from local Polish folk ensembles “Resovia Saltans” and “Bandoska” in Rzeszow, Poland, and the post-secondary school of cultural animators students in Krosno, Poland. The investigation was performed in the evenings, before the dancers’ training, in the training room by a physiotherapist. Study procedures included a self-administered questionnaire and a physical examination of the sagittal spine curvatures and ROM. The questionnaire included questions about age, body weight and height, dancing experience in years, training intensity (number of training hours per week), the occurrence of chronic low back pain and its intensity (VAS). As the nature of pain makes objective measurement impossible, simple, one-dimensional tools such as numeric rating scales (NRS) and visual analogue scales (VAS) were introduced to the clinical practice [29]. The VAS is a valid and reliable measure of chronic and acute pain intensity that is widely used by clinicians [30]. Next, we assessed the sagittal curvatures of the spine using an electronic inclinometer (smartphone Xiaomi Redmi 11S 5G). As smartphones are objects of daily living that are small, cost-effective, easy to use, and include an inertial motion unit composed of 3D accelerometers, gyroscopes, and digital magnetometer sensors, mobile phone-based measurements became more and more common in clinical settings and research as they are reliable and valid for non-invasive clinical measurements of mobility and joint positioning [31,32,33,34]. The sagittal spine curvature measurements were performed in a standing upright position with straight lower limbs according to the previous methodologies [35,36,37]. The assessor performed the following curvature measurements of the spine: sacral inclination, lumbar lordosis and thoracic kyphosis. The sacral inclination was measured by placing the upper beam of the inclinometer in the middle of the line between the posterior superior iliac spines. The lumbar lordosis was measured as a sum of the angles between the middle of the line between the posterior superior iliac spines and the thoracolumbar junction. The thoracic kyphosis was calculated as the sum of angles between the thoracolumbar junction and the middle of the line between the angulus superior of the scapulas [37]. This assessment has been shown in Figure 2.

The assessment of spinal flexion was performed using Otto’s and Schober’s tests. The Schober’s test evaluates the lumbar spine ROM in flexion in a standing upright position and is characterized by moderate validity but excellent reliability [38]. The measurement started by finding the posterior superior iliac spine on both sides and marking the midpoint of the line connecting both points. Using the tape, the assessor measured the distance of 10 cm upward and marked the second bony point. Then, the participants were instructed to flex forward slowly as far as possible with straight lower limbs. The measurement between the two marks was repeated and the difference between the two measurements indicated the outcome of the lumbar spine flexion. The greater difference between the two measurements indicates the greater lumbar spine mobility [39,40,41]. The flexion of the thoracic spine was assessed using the Otto’s test. Similarly to the previous test, the assessor marked two points on the participants’ spine; the first one on the participants’ spinal process of Th1 vertebra, and the second one 30 cm downward using a measuring tape. The participants were instructed to bend forward slowly with straight lower limbs as far as possible. The difference between the two measurements showed thoracic spine flexion; greater difference between the two measurements indicated greater mobility [42,43,44]. The study protocol was approved by the Bioethics Commission at the Medical University of Lublin. All study procedures were carried out in compliance with the Declaration of Helsinki and all the study participants gave their written informed consent before the start of the investigation.

### 2.3. Statistical Analysis

The collected data was analyzed using Statistica 13.3. Firstly, the normality of distribution was assessed using the Shapiro-Wilk test. Because the analyzed data was not distributed normally, non-parametric tests were performed. The differences between the study groups in quantitative data were assessed using the Mann–Whitney U-test (when comparing dancing experience and training intensity between folk and ballroom dancers) or the Kruskall–Wallis test (when comparing anthropometric measurements, spinal mobility, depth of spinal curvatures, and low back pain intensity between folk and ballroom dancers, and non-dancers). The associations between qualitative data were assessed using the chi-square test. Quantitative data were presented as mean, standard deviation, min, max, and median (Q1–Q3), and qualitative data were presented as n (%). The correlation between the two parameters was assessed and presented as Spearman’s rank correlation coefficient (r). The level of statistical significance was assumed if *p* < 0.05.

## 3. Results

The study groups did not differ significantly in age, height, body mass, BMI, and distribution of the subjects in gender. Also, no statistically significant differences were found in dancing experience and dance training intensity between the ballroom and folk dancers. Detailed study groups’ characteristics have been shown in Table 1.

The evaluation of spinal mobility and spinal curvatures in the examined groups showed a statistically significant disparity in thoracic kyphosis with a significance level of *p* = 0.02. The post-hoc analysis revealed that a significant difference in thoracic kyphosis appeared between FD and BD groups. The median thoracic kyphosis in the FD group was 40 (37–47.8) degrees. In contrast, the BD group demonstrated a median thoracic kyphosis of 33 (29–38) degrees. There were no other significant differences between groups in other sagittal spinal curvatures. Analysis of data in the studied groups indicated median values in the Otto’s test for FD as 2 cm (1–3), BD as 2 (0.5–2.5) cm, and ND as 2.5 (1.5–3) cm. Median values in the Schober’s test were as follows: FD = 5 (3–6) cm, BD = 6 (5–6) cm, and ND = 5 (4–6) cm. Nonetheless, no statistically significant differences were detected in Otto’s and Schober’s mobility tests (*p* > 0.05). Detailed data are presented in Table 2.

The analysis of the occurrence of chronic low back pain revealed no statistically significant differences among the study groups. However, the results of the BD group, at a level of 66.7%, suggest a higher prevalence compared to the other groups. The frequency of chronic low back pain occurrence in each group is presented in Table 3.

The interpretation of the results regarding the intensity of low back pain in different study groups revealed a lack of statistical significance. The lowest median pain score was recorded in the BD group at the level of 3 (3–3.5), while the FD and ND groups presented median scores of 6 (4–7) and 5.5 (4–7), respectively. Detailed data can be found in Table 4.

The evaluation of the results concerning the correlation between the intensity of lower back pain and spine mobility in different study groups revealed a very strong negative correlation in the ND group at the level of Th ROM: r = −0.95, *p* = 0.003. No significant differences were observed in the other groups. The obtained results are presented in Table 5.

Data analysis in the FD group revealed a correlation coefficient between dancing experience and BMI at a level of 0.67, indicating a very strong positive correlation at the level of *p* = 0.002. In the BD group, the *p*-value for the correlation between low back pain intensity and training intensity was observed at 0.07, which is close to but slightly above the assumed significance level. This suggests that there may be a trend, but it was not statistically significant in this analysis. Detailed data are presented in Table 6.

## 4. Discussion

In this study, we evaluated the impact of practicing Polish folk and ballroom dancing on the dancers’ body posture as well as spine mobility and low back pain. We also attempted to compare those parameters between these groups and look for correlations between pain intensity and body posture. As mentioned above, Polish folk and ballroom dances differ significantly. Polish folk dance depends strictly on the geographical region it was created and because of that, it varies in style and specific dance elements. In general, Polish folk dance is dynamic with characteristic rhythm of the music placing a heavy workload on dancers by covering large distances on stage. Common folk dance elements include paired and chain dancing [45]. Polish folk dance is more squat and requires a lot of jumping, kneeling, spinning, and maintaining the immobile trunk. On the other hand, ballroom dance also varies depending on the specific categories (Standard or Latin), both of them are different in style and dynamic. In general, ballroom dancing requires high movement dynamics and a greater hyperextension of the spine and rotation movements of the trunk and pelvis. The characteristic and often used closed-hold position, in which ballroom dancers face each other, the hands, arms, thighs, and pelvis are connected. Despite that, dancers move quickly and smoothly. During the hold, while male dancers maintain the trunk in an upright position, female dancers perform hyperextension and lateral flexion in the trunk and neck increasing the dance aesthetics [46]. The evaluation of spinal mobility and spinal curvatures in the examined groups revealed significant differences only in thoracic kyphosis angle between folk and ballroom dancers. The median thoracic kyphosis was greater in folk dancers compared to ballroom dancers and non-dancers. There were no other significant differences between groups in other sagittal spinal curvatures and spine mobility. Changes in the characteristic position and main movements between folk and ballroom dances may indicate changes in posture and low back pain. Swain et al., analyzed spine posture, maximum ROM, and movement asymmetry of female professional and student dancers aged 15 and above from both classical ballet and contemporary dance styles using a nine-camera three-dimensional motion analysis system. In contrast, the obtained results were somehow different from ours, as female dancers were characterized by a flatter spine posture and increased spine ROM compared to non-dancers [47]. We assume that this difference may result not only from different dance styles and the younger age of participants but also from the fact that in our study we included both male and female dancers. McMeeken et al., recruited 41 dancers aged 10–25 and used a computer-based analysis of videotape records examining sagittal standing posture and thoracolumbar flexion-extension mobility. Similarly to Swain’s study, the dancers, compared to non-dancers, were characterized by straighter standing postures and greater thoracic and lumbar sagittal excursions [48]. The different results may also be affected by the younger age of participants and different dance styles. In our study, the prevalence of chronic low back pain was highest in ballroom dancers (67%) compared to folk dancers and non-dancers. Interestingly, the median pain intensity was lowest in ballroom dancers and greatest in folk dancers; these differences were, however, insignificant. Nevertheless, the high prevalence of low back pain is a serious health problem and it may be caused by either poor technique, as in the case of forcing turnout, or poor core strength which may lead to further musculoskeletal pathologies [49]. Furthermore, low back pain in dancers is also associated with altered motor control of the lumbopelvic region [50]. Henn et al., conducted a systematic review analyzing twenty-five ballet, five modern, and three hip-hop articles. The results suggest that the prevalence of low back pain is relatively high in ballet dancers not as likely in modern dancers, and possibly a higher risk in hip-hop dancers. The author paid attention, however, to not enough high-quality research on the subject [51]. In our study, we also found no correlation between low back pain and spine curvatures and ROM in both folk and ballroom dancers, however, we found a very strong and negative correlation between thoracic spine flexion and the pain intensity in non-dancers. Similar results were obtained by McMeeken et al. [48], but not Swain et al. [47]. We suspect that dancing and dance-specific warm-up exercises may improve thoracic flexion and compensate for low back pain caused by a limited thoracic ROM. As known, limited flexibility is associated with low back pain and exercise programs are effective in reducing low back pain by improving muscle strength and flexibility [52,53]. It is, therefore, important to encourage people at risk of developing low back pain to exercise regularly and include ROM exercises in their daily routines. The analysis also revealed that only in folk dancers, but not in ballroom dancers, the BMI correlates very strongly and positively with the dancing experience. This is quite an interesting result and should be the subject of future research considerations taking into account folk dancers’ body composition to investigate whether this very strong and positive correlation between BMI and dancing experience is related to developed muscle or fat mass [54]. From a practical and clinical point of view, understanding the relationship between practicing particular forms of dance and body posture, low back pain and spine mobility may allow dance instructors to optimize the training process and sensitize them to specific musculoskeletal overuse pathologies, as well as reduce the risk of future musculoskeletal injuries. The main limitation of our study is that the sample size is small, thus our results should be treated with caution, however, this is due to limited access to folk dancers at a certain age, as there are no special schools that would educate folk dance professionals as in ballet dances; nonetheless, the sample size in our study is similar to other dance-related studies [55]. Another limitation is a lack of confirmation whether study participants with low back pain were or were not currently receiving treatment such as painkillers or physiotherapy.

## 5. Conclusions

The prevalence of low back pain in dancers is higher than in non-dancers. There are no differences in low back pain occurrence and pain intensity between folk and ballroom dancers, and non-dancers. Interestingly, only in non-dancers, there was a very strong and negative correlation between thoracic flexion and the intensity of low back pain. We suspect that dancing and dance-specific warm-up exercises may improve thoracic flexion and compensate for low back pain caused by limited thoracic ROM. The ballroom dancers tend to have a flatter spine posture in the thoracic area, while folk dancers seem to have more flexed posture in this area. Further research based on a larger study population should be conducted to draw solid conclusions. Due to the high prevalence of low back pain among folk and ballroom dancers, it is recommended to further investigate the low back pain risk factors in these groups. It is also recommended to increase awareness among dance instructors to optimize the training process and sensitize them to specific musculoskeletal overuse pathologies, as well as reduce the risk of future musculoskeletal injuries.

## Figures and Tables

**Table 1:** Study groups’ characteristics.

Variable	Group	Female			Male		Total		χ^2^	*p*
		n		%	n	%	n	%		
Gender	FD	13		68.42%	6	31.58%	19	37.25%	0.85	0.65
	BD	8		53.33%	7	46.67%	15	29.41%		
	ND	11		64.71%	6	35.29%	17	33.33%		
		Mean	SD	Min	Max	Q_1_	Me	Q_3_	H	*p*
Age	FD	23.7	3.88	21	32	21	23	28	4.36	0.11
	BD	25.4	2.77	19	30	24	25	29		
	ND	25.4	3.04	18	30	24	25	28		
Body mass [kg]	FD	64.4	10.53	51	78	55	60	77	2.73	0.26
	BD	69.4	11.03	52	84	61	65	80		
	ND	67.94	17.20	51	115	56	63	70		
Height [cm]	FD	171	9.89	155	192	164	169	178	4.55	0.1
	BD	175	7.83	159	189	169	175	182		
	ND	169	6.18	160	180	160	169	174		
BMI	FD	22.04	2.42	18.78	28.28	20.31	22.06	23.29	0.65	0.72
	BD	22.47	1.86	18.72	25.25	20.89	22.03	24.34		
	ND	23.64	5.55	18.37	41.73	20.32	22.75	23.46		
		Mean	SD	Min	Max	Q_1_	Me	Q_3_	z	*p*
Dancing experience [years]	FD	8.55	3.51	2	15	6	10	10	−1.6	0.11
	BD	6.67	3.02	2	12	4	7	9		
Training intensity [h/week]	FD	4.2	1.12	2	7	3	4	7	−0.6	0.58
	BD	3.9	1.34	2	6	3	4	6		

FD—Folk dancers; BD—Ballroom dancers; ND—Non-dancers; *n*—Number of subjects; SD—Standard deviation; Q_1_—Lower quartile; Me—Median; Q_3_—Upper quartile; χ^2^—chi-square test value; H—Kruskal–Wallis test value; z—Mann–Whitney U test value; *p*—*p* value.

**Table 2:** Spine mobility and depth of spinal curvatures in study groups.

Group	Mean	SD	Min	Max	Q_1_	Me	Q_3_	H	*p*
Segmental mobility of the spine									
Otto’s Test [cm]									
FD	2.29	1.14	1	5.5	1	2	3	2.33	0.31
BD	1.93	1.67	0.5	7	0.5	2	2.5		
ND	2.29	1.18	0	4	1.5	2.5	3		
Schober’s Test [cm]									
FD	4.61	1.71	2	7	3	5	6	2.70	0.26
BD	5.6	1.18	2.5	8	5	6	6		
ND	5.18	1.69	4	9	4	5	6		
Depth of spinal curvatures									
Sacral inclination [degree]									
FD	25.5	3.64	17	30	23	27	28	0.40	0.82
BD	24.79	7.41	10.5	35	18	25	29		
ND	26.14	7.79	9.5	40	22.5	27	30		
Lumbar lordosis [degree]									
FD	39.05	5.92	31	49.2	34	37	45	0.49	0.78
BD	37	3.82	31	43.5	34	37	39		
ND	38.62	7.44	27	53.7	35	37	40		
Thoracic kyphosis [degree]									
FD	41.57	7.53	29	55.5	37	40	47.8	7.84	0.02
BD	34	6.57	22	47	29	33	38		
ND	40.36	8.74	26	57.3	35.3	39.6	44.8		

FD—Folk dancers; BD—Ballroom dancers; ND—Non-dancers; SD—Standard deviation; Q_1_—Lower quartile; Me—Median; Q_3_—Upper quartile; H—Kruskal–Wallis test value; *p*—*p* value.

**Table 3:** The occurrence of chronic low back pain in study groups.

Group	Yes		No		Total		χ^2^	*p*
	n	%	n	%	n	%		
Chronic low back pain							3.49	0.17
FD	8	42.1%	11	57.9%	19	37.3%		
BD	10	66.7%	5	33.3%	15	29.4%		
ND	6	35.3%	11	64.7%	17	33.3%		

FD—Folk dancers; BD—Ballroom dancers; ND—Non-dancers; *n*—Number of subjects; χ^2^—chi-square test value; *p*—*p* value.

**Table 4:** Low back pain intensity in study groups.

Group	Mean	SD	Min	Max	Q_1_	Me	Q_3_	H	*p*
Low back pain intensity									
FD	5.75	2.21	3	8	4	6	7	2.83	0.24
BD	3.25	0.5	3	6	3	3	3.5		
ND	5.5	2.08	3	8	4	5.5	7		

FD—Folk dancers; BD—Ballroom dancers; ND—Non-dancers; SD—Standard deviation; Q_1_—Lower quartile; Me—Median; Q_3_—Upper quartile; H—Kruskal–Wallis test value; *p*—*p* value.

**Table 5:** The correlation between low back pain intensity and spine mobility and curvatures depth.

Variable	FD		BD		ND	
	r	*p*	r	*p*	r	*p*
Thoracic ROM	−0.3	0.47	−0.11	0.78	−0.95	0.003
Lumbar ROM	−0.44	0.28	0.23	0.55	0.21	0.69
Sacral inclination	0.23	0.58	−0.42	0.27	−0.64	0.17
Lumbar lordosis	−0.04	0.92	−0.4	0.29	0.03	0.95
Thoracic kyphosis	0.4	0.33	0.37	0.33	0.03	0.95

FD—Folk dancers; BD—Ballroom dancers; ND—Non-dancers; r—Spearman’s rank correlation coefficient; *p*—*p* value.

**Table 6:** Correlation between low back pain intensity, BMI and dancing experience and training intensity.

Variable	FD		BD	
	r	*p*	r	*p*
Low back pain intensity				
Dancing experience	0.06	0.88	0.52	0.15
Training intensity	−0.21	0.62	−0.64	0.07
BMI				
Dancing experience	0.67	0.002	−0.17	0.56
Training intensity	0.26	0.27	−0.2	0.46

FD—Folk dancers; BD—Ballroom dancers; r—Spearman’s rank correlation coefficient; *p*—*p* value.

## Data Availability

The datasets used and/or analyzed during the current study are available from the corresponding author on reasonable request.
